# Vascular Permeability in Diseases

**DOI:** 10.3390/ijms23073645

**Published:** 2022-03-26

**Authors:** Jean-Luc Wautier, Marie-Paule Wautier

**Affiliations:** Faculté de Médecine, Université Denis Diderot Paris, 75013 Paris, France; mpwautier@hotmail.com

**Keywords:** vascular permeability, endothelial cells, endothelial junctions, nitric oxide, prostacyclin, vascular endothelial cell growth factor, cerebral edema, macular edema, diabetic vasculopathy

## Abstract

Vascular permeability is a selective mechanism that maintains the exchange between vessels, tissues, and organs. The regulation was mostly studied during the nineteenth century by physiologists who defined physical laws and equations, taking blood, tissue interstitial, and oncotic pressure into account. During the last decades, a better knowledge of vascular cell functions and blood-vessel interactions opens a new area of vascular biology. Endothelial cell receptors vascular cell adhesion molecule (VCAM), intercellular cell adhesion molecule (ICAM), vascular endothelial growth factor receptor (VEGFR-2), receptor for advanced glycation end products (RAGE), and mediators were identified and their role in homeostasis and pathological situations was described. The molecular differences of endothelial cell junctions (tight, gap, and adherens junctions) and their role in vascular permeability were characterized in different organs. The main mediators of vasomotricity and permeability, such as prostaglandins, nitric oxide (NO), prostacyclin, vascular growth factor (VEGF), and cytokines, have been demonstrated to possess major functions in steady state and pathological situations. Leukocytes were shown to adhere to endothelium and migrate during inflammatory situations and infectious diseases. Increased vascular permeability is linked to endothelium integrity. Glycocalyx, when intact, may limit cancer cell metastasis. Biological modifications of blood and tissue constituents occurring in diabetes mellitus were responsible for increased permeability and, consequently, ocular and renal complications. Vascular pressure and fluidity are major determinants of pulmonary and cerebral edema. Beside the treatment of the infectious disease, of the blood circulation dysfunction and inflammatory condition, drugs (cyclooxygenase inhibitors) and specific antibodies anti-cytokine (anti-VEGF) have been demonstrated to reduce the severity and the mortality in diseases that exhibited enhanced vascular permeability.

## 1. Introduction

During the nineteenth century, physiologists described the forces responsible for fluid movement between blood vessels and their surrounding tissues. In France, J.L. Poiseuille published, in 1839, a formula that was then known as the Poiseuille law, or Hagen–Poiseuille [1]. He had several discussions with M.J. Muller on the realty of the mechanism. In Great Britain, E.H. Starling defined the forces involved in homeostasis tissue pressure (Pt) plasma colloidal osmotic (πp) capillary pressure (P_c_) and tissue colloidal osmotic pressure (πt) [2]. The correlation between hydrostatic capillary pressure and rates of fluid movement and absorption through the vessel wall was formulated as an equation: P_c_ − Pt = πp − πt. A revised equation was written by E.M. Landis: J_V_ = K [(P_c_ − P_i_) − (Π_p_ − Π_i_)] = K (ΔP − ΔΠ), where symbol J_V_ is the fluid flow rate, K is a microvascular filtration coefficient, P_c_ is microvascular hydrostatic pressure, P_i_ interstitial fluid hydrostatic pressure, Π_p_ microvascular plasma oncotic pressure, Π_i_ interstitial fluid oncotic pressure, and ΔP and ΔΠ are the differences in hydrostatic and oncotic pressures across microvascular wall.

Microvascular walls are permeable to proteins, but homeostasis is maintained by a low filtration and occurs differently, according to the tissues and organs. G. Grotte proposed that separate pathways existed, including small and large pores [3]. Different theories are proposed by J.R. Levick and C.C. Michel [4], involving the role of glycocalyx and the endothelial cell tight junctions.

After the historical debates, mostly supported by the physiologists, a new approach was possible, following the progress made in the understanding of the vascular cell biology, as well as the discovery of the various mediators that now define homeostatic vascular function and permeability.

We will focus our review on the biological aspects of the vessel permeability and some diseases in which the alteration of the vessel permeability is responsible for major complications. Since we have substantial research in diabetes mellitus patients, in animal experiments and in vitro models, we will further detail the mechanisms involving advanced glycation end products (AGE), as well as the consequence of AGE binding to receptor for advanced glycation end products (RAGE), in the section dedicated to vascular permeability in diabetes mellitus.

In healthy conditions, human organs and cells are connected directly or individually by vessels of different morphologies and functions. These vessels provide blood to tissues and maintain physiological functions. Blood is composed of cells and fluids; the circulation is dependent on heart functions. The exchange between blood components and tissues is a complex system, essential for maintaining homeostasis. Blood vessels are lined by endothelial cells, which represent a large surface of exchange (approximately 7000 m^2^). Endothelium is composed of a monolayer of endothelial cells, limiting the vessel wall in the inner part, and in close contact with the subendothelial matrix linked to different types of collagens and smooth muscle cells, according to the type of vessel. Endothelial cells are different, according to the anatomical location and type of vessel, artery, vein, or micro vessel. They have different properties, in terms of reactivity to stimuli; in summary endothelium is a dynamic surface.

## 2. Mechanism of Permeability

### 2.1. Definition

The historical definition of vascular permeability implies the vascular sieving of solutes and molecules, which is observed in normal, stable conditions; it can be modulated and altered in diseases. Endothelial cells and, to a lesser extent, vascular mural cells control vascular permeability. Water and solute exchange across the walls occurred mainly in the microcirculation. Three molecules present in plasma are considered to be of a major importance in the balance between blood and interstitial pressure: albumin, globulins, and fibrinogen [5]. Inflammatory stimuli increase the permeability of the vessel, which induces the extravasation of molecules larger than 40 kDa, including plasma proteins. Different mechanisms are involved in the process of the passage of the molecules through the vascular endothelium. It can be divided into three steps:

- The glycocalyx covered the endothelial cell surface, and it is in contact with blood components.
- The transendothelial trafficking is energy dependent pathway.
- Opening of cell/cell modification.

### 2.2. Endothelial Cell Junctions

**Figure 1:**
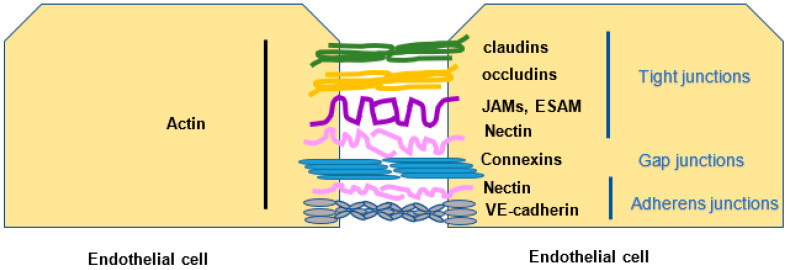
Endothelial cell junctions. Endothelial cells linked one to the other by different types of junctions form a barrier. Tight junctions: claudins, occludins, junction adhesion molecule (JAMs), endothelial cell-selective adhesion molecule (ESAM), and nectin. Gap junctions: connexins. Adherens junctions: nectin, VE-cadherin.

Endothelial cell junctions are divided into tight, gap, and adherens junctions. Different molecules participate in tight junctions (claudins, occluding, junction adhesion molecules, endothelial cell-selective adhesion molecules, nectin) that bind to actin. Connexins are the constituent of gap junctions. Six connexins (CXs) form a connexon or hemichannel, and the docking of two connexons results in a full gap junction channel Cxs, which are expressed in virtually all tissues and cell types, including the endothelial cells (EC) and smooth muscle cells (SMC) of blood and lymphatic vessels [6]. Adherens junctions (nectin, VE-cadherin) are linked to the actin and vimentin of the adjacent EC (Figure 1).

**Figure 2:**
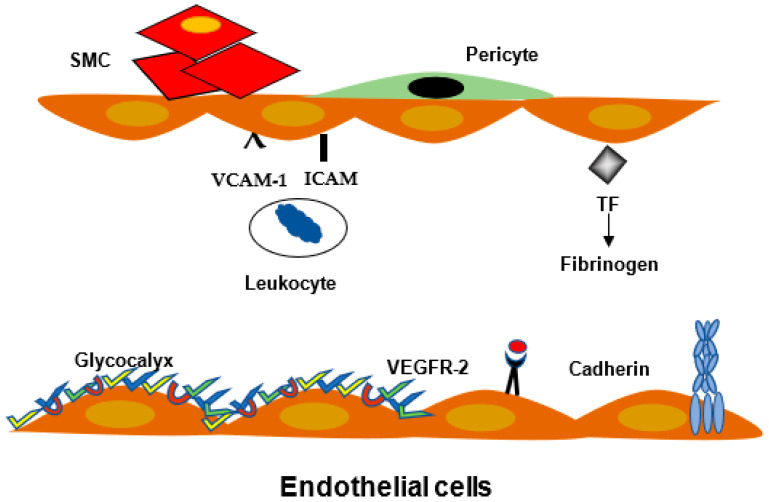
Vessel wall and glycocalyx. EC are in close proximity to smooth muscle cells (SMC) and pericytes, which, via mediators, have a continuous cross talk. EC are covered on the inner site by the glycocalyx. They expose the tissue factor, which can initiate coagulation. The receptors involved in leukocyte adhesion, such as the vascular adhesion molecule (VCAM) and intercellular adhesion molecule (ICAM), are modulated by cytokines tumor necrosis factor α (TNFα), interleukin-1 (IL-1), interferon γ (IFNγ). The vascular endothelial growth factor (VEGF) and receptor VEGFR-2 induce VE-cadherin phosphorylation and junction opening.

The endothelial glycocalyx layer, found on the luminal surface of all endothelial cells, contributes to the regulation of the permeability barrier, formed by the vessel wall. The glycocalyx is not uniform (Figure 2). It is composed of a two-layer fiber matrix model, which suggests a dense 200 to 300 nm mesh-like inner layer, rich in proteoglycans, covalently bound to the endothelial cell membrane and adherent glycosaminoglycans, including long chains of hyaluronan (HA), and an outer, more porous, gel-like layer up to 1 mm thick, including adsorbed plasma proteins [7]. Human diseases, in which glycocalyx is damaged, are also associated with permeability alterations.

### 2.3. Leukocyte Migration and Inflammation

During immune response, or in inflammatory conditions, leukocytes transmigrate through the endothelium. Leukocyte adhesion to endothelium is mediated by different adhesion molecules. Leukocyte extravasation migration is an essential step in inflammatory conditions, such as infectious and vascular diseases. The interaction between blood leukocytes can be divided in several steps, i.e., rolling, stable adhesion, and extravasation; they are mediated by different cytokines, such as tumor necrosis factor α (TNFα), interferon γ (INFγ), and interleukin-1 β (IL1β). Activated endothelial cells expressed E selectin and vascular cell adhesion molecule (VCAM). In parallel, intercellular cell adhesion molecule (ICAM) expression can be augmented by cytokines. After rolling, the leukocyte CD18 family molecules bind to their respective ligands. Leukocyte integrins β2 (CD18) α subunits (CD11a, CD11b, CD11c, and CD11d) bind to EC adhesion molecules ICAM VCAM on the endothelial site [8] (Figure 2).

Mechanical forces modify cell functions. The component of the hemodynamic forces, also known shear stress, is a modulator of vascular cell functions. Integrins participate in the attachment of cells to the extracellular matrix and attraction between the cell Rho family. GTPases have distinct functions in regulating actin-based cytoskeletal structures [9]. Shear stress activates integrins, VEGF receptor (Flk-1), and G protein-coupled receptors. Alpha and Beta integrins, in response to shear stress, transactivate the VEGF receptor, which binds casitas B-lineage lymphoma (Cbl) and regulates the Kb protein kinase inhibitor. These modifications regulate the NF-kB pathway [10].

During myocardial infarction, vessel occlusion and poor blood flow produce an increase of coronary vessel permeability. Polynuclear neutrophils infiltrate the infarcted myocardium and contribute to tissue damage, releasing reactive oxygen intermediates, resulting from NADPH oxidase activation. In parallel, myeloperoxidase, released from leukocytes, is a marker of risk [11,12].

### 2.4. Blood Brain Barrier

Arterioles, venules, and capillaries, formed the brain microvascular network, and the whole, constitutes the blood brain barrier (BBB). The BBB is formed by endothelial cells of the cerebral capillaries. The cerebrospinal barrier is constituted by the epithelial cells of the choroid plexus. The avascular arachnoid epithelium, considered as the third barrier, underlines the dura mater [13].

Pericytes are present in different organs; in the central nervous system, they are found in a multicellular structure, named the neurovascular unit [14]. When the BBB is altered, it can result in an increased permeability and, as in other organs, endothelial cell activation and expression of receptor enhanced leukocyte adhesion and immune reaction [15]. Pericyte endothelial cell interactions may be modulated by platelet-derived growth factor (PDGF β), transforming growth factor β (TGF β), and angiopoietins. Pericytes, present in the retina and brain (Figure 2), regulate neoangiogenesis. In diabetic patients, pericytes participate in the regenerative process [16].

## 3. Modulation of Vascular Permeability

Vasodilatation can be induced by several mediators: acetylcholine, ATP, adenosine, bradykinin, histamine, and shear stress, which can activate the endothelial nitric oxide synthase (eNOS) and cyclo-oxygenase (COX) pathways that lead, respectively, to nitric oxide (NO) [17,18] and prostacyclin (PGI 2) formation. NO and PGI 2 act through a second messenger; NO uses cyclic guanosine monophosphate (CGMP), while PGI 2 effects are mediated by cyclic adenosine monophosphate (CAMP). Another step occurs in smooth muscle cells, associated with a decrease in intracellular Ca and phosphatase activity (myosin light chain phosphatase).

### 3.1. Prostaglandins

**Figure 3:**
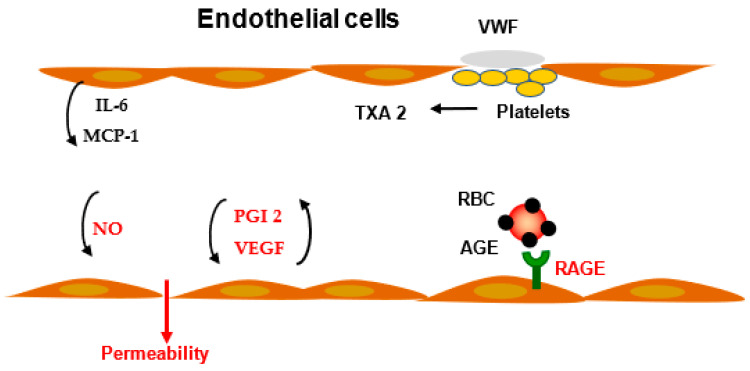
Modulation of vascular permeability and inflammation. Activated EC produced and released interleukin-6 (IL-6) and macrophage (monocyte) protein-1 (MCP-1) in inflammatory conditions. Advanced glycation end products (AGE), present on protein or red blood cell (RBC), bind to a specific receptor (RAGE), inducing a cascade of reactions, resulting in an increased vascular permeability. Nitric oxide (NO) is one of the mediators for vascular tone and vascular permeability. Prostacyclin (PGI 2) modulates vascular pressure and permeability. Activated platelets, after adhesion to the matrix and von Willebrand factor (VWF), release thromboxane A2 (TXA2), which contributes to permeability regulation.

Prostaglandins have a major role in the control of inflammatory processes. As an example, activation of the PGE2-EP2 or EP4 receptor signal induces vasodilatation. Prostacyclin has a potent role in vasorelaxation. Blockade of cyclooxygenase reduced the arachidonic acid formation and, consequently, prostaglandin formation. In the vessel wall, PGI 2, a vasodilatator, while in platelets thromboxane A2 and B2, a vasoconstrictor, are reduced. The balance between thromboxane and prostacyclin formation is crucial for the homeostasis of the vascular tone [19] (Figure 3).

### 3.2. VEGF

Vascular endothelial growth factor (VEGF) induces the stimulation of the c-Src family kinases, which successively provokes Vav2, Rac GTPases, and p21-actvated kinases (PAK) activation, leading to VE-cadherin phosphorylation and opening endothelial cell junctions. In addition to the effect of the Src family kinases (SFKs) on intercellular junctions, they can affect permeability through the regulation of focal adhesions [20] (Figure 3). Specialized subcellular structures mediate endothelial cell attachment: claudins, occludins, junction adhesion molecule (JAMs), endothelial cell-selective adhesion molecule ESAM, nectin, connexins, and VE-cadherin. Linkage to actin-related molecules plays a major role in the control of vascular permeability. Focal adhesion kinase (FAK) appears to be involved in both increasing or decreasing the endothelial permeability elicited by stimulation with the protease activated receptor 1 or sphingosine-1-phosphate receptor 1, respectively. The c-Src family kinases mediate vascular leakage, following stimulation by lipopolysaccharide (LPS) and VEGF. Lyn kinase, in contrast to other Src family kinases, strengthens endothelial junctions and, thereby, restrains the increase in vascular permeability. Lyn KO mice had increased vascular permeability and lethality, in response to LPS, compared with wild-type. Oppositely, when only Lyn is decreased, it did not affect LPS-induced endothelial permeability. Thus, in contrast to c-Src, which increases vascular permeability, in response to stimuli, Lyn stabilizes endothelial junctions through phosphorylation of FAK [21].

## 4. Vascular Permeability in Diseases

Vascular permeability, in various circumstances, can be modified. Hypothyroidism endothelium-dependent dilation is impaired but expression of mRNA for nitric oxide synthase is unchanged in muscles isolated from rats with thyroid dysfunction [22]. The elevated plasma level of homocysteine is associated with various vascular complications. Homocysteine limiting inhibition of metalloproteinases causes changes of the matrix of blood brain barrier and, consequently, the permeability [23,24].

### 4.1. Pulmonary Edema

Hemostasis of lung fluid is dependent upon endothelial cell barrier function. Increased flux of fluid and/or plasma-rich protein across endothelium of pulmonary capillaries results in pulmonary edema. Alteration of endothelial cell junctions VE-cadherin, associated with α, β catenin, and p120-catenin, leads to high permeability of pulmonary capillaries [25]. Pulmonary edema can be provoked by pathogens and inflammatory cells, mostly leukocytes and inflammatory mediators. Alternatively, left heart failure, high altitude, and high pulmonary pressure can be responsible of stress failure [26]. The mechanism resulting in the breakdown of the endothelial barrier function can be induced by pressure increase and endothelial cell activation. Lung vascular permeability can be increased by extracellular matrix alteration, for example, by in vitro modification of collagen crosslinking [27].

A newly described component, Piezo 1, a transmembrane molecule, can modulate the signaling pathways modifying membrane tension. It can modify vascular pressure and disturb endothelial adherens junctions. Piezo 1 is a potential target for pharmacological agents in the prevention of pulmonary edema [28]. 

Urokinase plasminogen activator (UPA) concentration is augmented in acute pulmonary lung injury. Mice deficient in UPA are protected against pulmonary edema induced by endotoxin; however, the mechanism involving UPA for the regulation of vascular tone and permeability in the lung is still not known [29].

### 4.2. Cerebral Edema

Various brain diseases, traumatic brain injury, and stroke can be associated to cerebral edema. It can be life-threatening, if not treated adequately and in a timely manner.

Osmotic agents, such as mannitol and hypertonic saline, are widely used to treat patients with cerebral edema. Cerebral edema may be divided into cytotoxic, ionic, and vasogenic edema.

The edema is related to the change of the brain blood barrier (BBB) permeability. Endothelial cells of the brain capillaries, in association with pericytes, astrocytes, and perivascular microglia, are essential for homeostasis in BBB. As observed in other organs or tissues VEGF is an important regulator of vascular permeability. VEGF R2 has a crucial role in VEGF function on permeability. VEGF and VEGF R2 expression was increased with augmentation of BBB permeability. The disruption of the tight junctions between endothelial cells leads to BBB breakdown.

Treatment by anti-VEGF antibodies can reduce edema. Hypoxia-inducible factor-1 (HIF-1) can affect VEGF expression. Tight junction components zonula occludens-1 (ZO-1) and Claudin-5 could be regulated and, consequently, modify BBB permeability. Hypertonic saline has been reported to reduce TNF-α and IL1-β and be beneficial for reducing inflammation and limiting the release of proinflammatory cytokines by astrocytes [30]

### 4.3. Infectious Diseases

In sepsis, elevated levels of TNF-α may participate in glycocalyx damage. In addition, a disintegrin metalloproteinase 15, heparanase, and matrix metalloproteinase 2/9 (MMP2/9) have been implicated in the degradation of glycocalyx [7]. 

SARS-CoV-2 proteins alter the barrier properties mediated by cell-junction proteins; 18 of 26 proteins of SARS-CoV-2, including nsp2, nsp5-c145a, and nsp7, affect the protein network regulating vascular functionality. The modulation of barrier functions can be measured via trans-epithelial–endothelial electrical resistance (TEER), which identifies changes in impedance values, reflecting the permeability of the cell monolayer [31]. In patients infected with SARS-CoV-2, fragmented vascular endothelial glycocalyx is elevated and may be an indicator of vascular complications [32].

SARS-CoV-2, after binding to the angiotensin-converting enzyme receptor, stimulates the formation of inflammatory cytokine (interleukin-6) and initiates coagulation, which leads to vascular thrombosis.

Anaphylaxis is a life-threatening type I allergic reaction, observed after contact to chemicals, venoms, bacteria, and/or virus components. The immunological-like reaction involved T cells, Th2 cytokines, and the production of IgE by B lymphocytes. The degranulation of basophils, provoked by IgE binding, results in the release of histamine, heparin, chymase, carboxypeptidase, TNFα, leukotriens, and VEGF. Several of these mediators, such as histamine and VEGF, increase vascular permeability [33]. 

Histamine and platelet activating factor (PAF) can stimulate NO synthesis and induce blood vessel dilatation, as well as the dysfunction of the endothelial barrier, by opening adherens junctions [5].

### 4.4. Cancer

Tumor metastasis is one of the main factors associated with high rates death in cancer patients. Cancer metastasis is favored by the enhancement of vascular permeability. Circulating tumor cells, moving in the microvasculature, tend to invade into stromal tissue at the location, where vascular permeability is enhanced. Interactions between tumor and endothelial cells are important steps mediated by different receptors or chemical structures. The extravasation of tumor cells is dependent upon vascular permeability [34]. At least two mechanisms are involved in trans-endothelial permeability, vesicle transport, and migration through endothelial cell junctions. A tripeptide derived from collagen (proline–glycine–proline) promotes VE-cadherin phosphorylation and enhanced vascular permeability. Tumor cells can bind to endothelial cells and induce EC necrosis via a TNF receptor family mechanism. Tumor cells can also release a large number of molecules that affect EC permeability [35].

Hyaluronic acid, heparan sulfate, and chondroitin sulfate, present in the glycocalyx, limit the access of circulating tumor cells to adhesion receptors, such as ICAM-1 or P selectin.

Serum amyloid A (SAA) is a family of reactants that can increase during the acute phase and serve as chemoattractant in proinflammatory phase (SAA3 and SAA1.1/2.1). SAA3, which is a major hepatic acute phase component in mice, is not produced in humans [36].

Thrombin, heparanase, and matrix metalloproteinase (MMP), produced in inflammatory conditions, degrade the glycocalyx component, favoring tumor cell access to the EC surface. The production of the tissue factor, by EC and/or macrophages, leads to factor X activation, which induces fibrin formation. Fibrinogen is highly represented in lung cancer and has been considered a permeability factor [37].

### 4.5. Diabetes Mellitus

In diabetes mellitus, classically divided into types 1 and 2, microvascular complications are responsible for abnormal permeability. The risk of cardiovascular complications is 2- to 4-fold higher in diabetic patients. Hyperglycemia is associated with a reduction of the glycocalyx via different interactions with the glycocalyx components. In addition, the shear stress-induced dilation is reduced by hyperglycemia, and several EC functions are directly altered by hyperglycemia, glycated proteins, or lipids [38].

The level of N epsilon-carboxymethyl lysine protein (CML) is increased in type 2 diabetic patients, and it also correlated with the augmented macrophage colony-stimulating factor (M-CSF) blood level [39]. A causal relationship may exist between these two parameters, since advanced glycation end products (AGE), formed either in vitro or patient AGE, enhanced M-CSF production by human umbilical vein endothelial cells (HUVEC). It has been previously established that, by binding AGE to receptor RAGE, this can produce an increased vascular permeability or the development of atherosclerotic lesion in animal models [40]. RAGE is expressed by endothelial cells, lymphocytes, and monocyte macrophages. RAGE ligands can bind to the different cell types and participate in inflammatory reactions [41].

The duo endothelial cell/monocyte functioned in harmony or opposite ways, depending on the organ and clinical situation, infectious diseases, inflammatory conditions, atherogenesis, and neoangiogenesis. 

Hyperpermeability is a precocious abnormality of diabetic vasculopathy. Endothelial cells in culture, when incubated with AGE proteins or glycated red blood cells, demonstrated an increased permeability to macromolecular tracers 125I albumin and 3H inulin, compared with endothelial cells incubated with normal proteins or red blood cells (RBC) from normal subjects. Membrane proteins of erythrocytes can be glycated: spectrin, band 3 transmembrane protein, and band 4–1 [42]. The glycation results in reduced RBC deformability and an increased adherence to endothelium [43]. When incubated with RBC from diabetic patients, anti-AGE antibodies or soluble RAGE (sRAGE) inhibit the enhanced adhesion to endothelium (Figure 3). The accelerated clearance of diabetic rat RBC, when infused in normal rats, is prevented by the co-infusion of the anti-RAGE antibody. These results support the concept that the abnormal adhesion of RBC, taken from diabetics, is mediated by the AGE present on RBC, as well as the RAGE expressed at the endothelial cell surface. The reduction of endothelial cell barrier function was inhibited by anti-AGE specific antibodies. In diabetic rats infused with soluble RAGE, hyperpermeability was corrected in the intestines and skin and suppressed by 90% in the kidney. According to reported experiments, reactive oxygen species formation is a likely means by which oxidative stress can increase vascular permeability by rapid changes in endothelial cell shape via calcium mediated pathways [44]. Increased reactive oxygen species (ROS) damage EC function and affect EC permeability. The best-known pathway of ROS generation involved NADPH oxidase [45].

As we previously showed, neoangiogenesis is impaired in animal models of diabetes, and collateral vessel development is significantly limited in diabetic patients [46]. Several factors may contribute to neoangiogenesis in diabetes. Glycation of basic fibroblast growth factor, with the intracellular sugars, fructose, and glucose-6-phosphate, reduced its high affinity heparin-binding capacity and mitogenic activity.

Glycated collagen induces premature endothelial cell senescence, as indicated by the appearance of senescence-associated β-galactosidase, increased cell size, and rate of apoptosis. This glycated collagen-induced senescence is associated with a decreased synthesis of NO. Premature senescence may contribute to diabetic vasculopathy. Enhanced inflammation of the vascular system is associated with an enhanced expression of cyclooxygenase-2 and PGE synthase-1 in human diabetic atherosclerotic plaques. AGE formation limits proteolysis of glycated proteins and, therefore, is deleterious for mechanical properties of the vessel wall.

We previously reported that, after 28 days, the ischemic/nonischemic leg angiographic ratio was decreased by 1.4-fold in diabetic animals, when compared with the control animals. In contrast, when diabetic mice were treated with aminoguanidine, the angiographic score was in the same range of the level observed in control animals. When the diabetic mice were treated by aminoguanidine, the AGE levels were decreased 4.2-fold, compared with untreated diabetic mice. When collagen is glycated, as it occurs during ageing, synthesis of NO is decreased. Blockade of AGE formation normalized impaired ischemia, which is probably mediated by restoration of matrix degradation processes [47].

Atherosclerosis and microangiopathy are observed in various pathologies, including diabetes mellitus. The potentiation of VCAM-1 expression may facilitate monocyte adhesion and extravasation. The statistically significant correlation between CML-protein blood level and M-CSF suggests that this mechanism may exist in diabetic patients. An inflammatory cascade of events may be initiated by AGE and involve chemotactic factors, leukocyte mediators [48], and prostaglandin modulation [46]. 

The relationships between CML, M-CSF, and VCAM-1 in diabetic patients with microangiopathy suggest that, beside the inflammatory reaction involved in the genesis of atherosclerotic lesions, endothelial activation can result, not only in vascular hyperpermeability, but also in the alteration of the microvasculature.

#### 4.5.1. Vascular Permeability in Kidney

One of the frequent complications in diabetes is diabetic nephropathy. Albuminuria is linked to glomerular dysfunction. Glomerulosclerosis, observed in diabetic animals, is associated with AGE deposition in mesangium and hyalinized and/or sclerotic lesions [49]. Levels of N epsilon-carboxymethyl lysine (CML) adducts are increased in soluble proteins and insoluble collagen in patients, with diabetes and renal impairment [50]. Endothelial cells are different, according to the organs. Hepatic sinusoidal endothelial cells and endothelium lining the glomerular tuft represent a permeable barrier with selective, different properties, which have major functions in homeostasis and organ failure in chronic and acute diseases [51].

RAGE overexpression enhanced glomerulosclerosis development in mice [52]. The pharmacological blockade of RAGE in db/db mice or genetic deletion of RAGE in mice with streptozotocin-induced hyperglycemia result in decreased albuminuria, mesangial expansion, and glomerulosis [53].

Methylglyoxal (MG)-derived hydroimidazolone MG-H1, N epsilon-carboxymethyl lysine, and glucosepane are quantitively important in AGE. The cellular proteolysis of AGE-modified proteins forms AGE-free adducts and glycated amino acids, which are cleared by the kidneys and excreted in urine. AGE-free adducts accumulate markedly in plasma, when the glomerular filtration rate declines. A key precursor of AGE is the dicarbonyl metabolite MG, which is metabolized by the glyoxalase 1 (Glo1) from the cytoplasmic glyoxalase system. An abnormal increase in MG dicarbonyl stress is a characteristic of chronic kidney disease (CKD) and driven by the down-regulation of renal Glo1, which increases flux of MG-H1 formation. 

In renal failure, peritoneal dialysis causes chemical peritonitis because of the limited biocompatibility of peritoneal dialysis fluids, which contain high glucose concentrations (up to 45 g/L) and, thus, the glucose-derived products that are the precursors for AGE. Due to the fact that RAGE is expressed on endothelial and mesothelial cells, the receptor may bind AGE present in patients or formed during peritoneal dialysis [54]. The binding of AGE to RAGE produces a local inflammatory reaction, likely as a consequence of vascular cell adhesion molecule-1 overexpression, leukocyte adhesion, and cytokine release [55].

#### 4.5.2. Retinopathy

The ocular complications are a hallmark of diabetic complications. Several decades ago, the deleterious effects of AGE formation in pig crystallin was observed in the pathogenesis of diabetic cataract [56]. One of the earliest changes observed in retinal micro vessels is the selective loss of intramural pericytes, which is a process that may be linked to the effects of AGE. AGE may induce apoptosis and necrosis in experimental models [55]. Macular edema is frequently observed in diabetic retinopathy. In reaction to hypoxemia-induced microvascular damage, the retinal epithelial and endothelial cells increase the production of VEGF and promote neoangiogenesis, which is a process that antibodies to RAGE prevent. Pyridoxamine, an inhibitor of AGE formation and lipoxidation end products, protects against diabetes-induced retinal vascular lesions [57]. The thiamine monophosphate derivative, benfotiamine, inhibits hyperglycemia-dependent pathways and NF-κB activation and prevents experimental diabetic retinopathy [58]. Beraprost sodium, a PGI 2 analog, has been reported to protect retinal pericytes from AGE-induced cytotoxicity, through its anti-oxidative properties; it was also previously shown to decrease vascular hyperpermeability in diabetic rats [59].

In patients with diabetes, mellitus diabetic macular edema and proliferative diabetic retinopathy (PDR) are the most frequent reasons for loss of vision. Macular edema causes the disruption of the inner blood-retinal barrier. Anti-VEGF was used to treat patients [60]. Diabetic retinopathy involves morphological and functional changes in the retinal capillaries. Basement membrane thickening, loss of pericytes, increased permeability, and vascular dysfunction are the prominent features. Diabetic macular edema is more commonly found in type 2 diabetes than in patients with type 1 diabetes.

On another hand, AGE can exert deleterious effects by acting directly to induce the cross linking of long-lived proteins, in order to promote vascular stiffness and, by interacting with receptor for AGE (RAGE), induce intracellular signaling, which leads to enhanced oxidative stress and the production of pro-inflammatory cytokines. Increased AGE accumulation has been found in cataract lenses in patients when ageing. Glycation of vitreal collagen fibrils can provoke the destabilization of the gel structure vitreous liquefaction and posterior vitreous detachment.

In diabetic patients, an increase in skin concentration of pentosidine is associated with the development of proliferative retinopathy [61]. The same holds true for 2-(2-fuoryl)-4(5)-(2-furanyl)-1H-imidazole (FFI), N-(epsilon) (carboxymethyl)lysine, which increases in parallel with the increasing severity of retinopathy [62].

### 4.6. Permeability and Atherogenesis

Current theory of atherogenesis opens a large role to lipids, stimulating M1 macrophage inflammatory response. Monocytes, recruited in the sub endothelium space, phagocyte lipids and produce inflammatory cytokines. Macrophages can stimulate angiogenesis, increase permeability, and augment inflammatory cell attraction.

In atherosclerotic plaque, defective endothelial junctions are associated with inflammation and a potentiation of VEGFA-VEGFR2 interactions [63]. Endothelium, with altered barrier function, facilitates LDL accumulation. The LDL passage is first limited by the glycocalyx then LDL transport through EC via transcytosis. The different types of dyslipidemia alter EC functions. Hyperlipidemic serum increases permeability of EC in culture. Atherosclerosis progression resulted from multiple interactions between macrophages, endothelial cells, smooth muscle cells, and matrix cells. Plaques that exhibited increased vascular permeability have a higher propension to thrombosis. 

Ultrasmall superparamagnetic iron oxide particles (UPSIOs) deposit in areas with abnormal permeability. UPSIOs are macrophage markers, not only in atherotic plaque, but also in inflammatory milieu.

Smooth muscle cells exhibited adhesion molecules and expressed tissue factors, which may lead to atherothrombosis. Evans blue deposition, used as permeability index, demonstrated that impaired endothelial permeability is associated with CLIO-CyAm7 (iron oxide nanoparticles) deposition. Impaired endothelial barrier function facilitates macrophage accumulation in the atheroma intima [64]. 

## 5. Conclusions, Treatment, and Perspectives

In developed countries, dial macular edema (DME) has now overtaken proliferative diabetic retinopathy (PDR) as the more common vision-threatening form of diabetic retinopathy, particularly among patients with type 2 diabetes. The incidence of visual impairment among people with diabetic retinopathy has halved, likely as a result of the lower risk of DME and PDR among patients with recently diagnosed diabetes [65]. Laser photocoagulation has been reported to be beneficial in treating PDR, but the evidence is moderate and based on old trials. Now pan-retinal photocoagulation is the most frequently used technique. The evidence of the efficacy and safety of anti-VEGF, for the treatment of PDR, is also limited. The results of new trials suggest that anti-VEGF can reduce the risk of intra-ocular bleeding in patients with PDR. Pars-plana vitrectomy is performed in advanced cases of PDR, alongside extensive tractional membranes, vitreous hemorrhage, and tractional retinal detachment [66].

Over the last decade, the intraocular administration of pharmacological agents (e.g., steroids and anti-VEGF agents) has been evaluated as a new treatment modality for DME and PDR. Although intraocular injections of long-acting steroids (e.g., triamcinolone) have demonstrated the ability to reduce DME and improve vision, these beneficial effects appear to be short-lived, and long-term visual outcomes were generally not better than conventional laser therapy [67]. In recent years, there has been a surge of clinical trials investigating the use of anti-VEGF therapy for DME [68]. These trials provide robust evidence that the intraocular administration of anti-VEGF agents is better than laser therapy, with regard to both preserving and improving vision for patients with DME. The rates of serious sight-threatening complications are acceptably low, as shown, not only in studies of patients with diabetic retinopathy, but also in those with age-related macular degeneration. Assuming equivalent effectiveness and similar safety profiles between bevacizumab and ranibizumab injections, the use of bevacizumab confers a greater value among the different treatment options for DME [69]. The systemic management of hyperglycemia, hypertension, and dyslipidemia remains the most commonly used strategy for preventing the development and progression of diabetic retinopathy [70].

The inhibition of the AGE–RAGE interaction and downstream effects may be a novel therapeutic approach. The restriction of AGE formation by antidiabetic treatment limited retinal vessel damage [71,72]. In experimental models, in vitro or in vivo, blocking RAGE (anti-RAGE, peptides) prevented endothelial cell alterations [73]. RAGE blood level appears to be correlated with the risk of microvascular complications. Therefore, modulation of the RAGE gene expression may, in the future, be a way to prevent microvascular lesions. 

A better understanding of abnormal vascular permeability may lead to a new, more specific therapeutical approach, according to the organ or disease. Piezo 1, a transmembrane molecule that can modify vascular pressure, is a potential target for pharmacological agents in the prevention of pulmonary edema

Anthocyanin, a soluble flavonoid, in experimental conditions, improves endothelial cell functions, reduces endothelin-1, and increases NO synthase activity. In parallel, luteomidin, an anthocyanidin, has anti-oxidant properties. The low plasma concentration of oral bioavailability remains the question of the efficacy. Anthocyanin (ACY), acute or chronic effect, is discussed. Cranberry juice has an acute, but not a chronic, benefit on flow mediated vasodilation. Alternatively, ACY may influence the arterial wall [74].

Natriuremic peptides may inhibit the renin-angiotensin system and have diuretic natriuremic and vasodilatory actions [75]. 

The important role of the glycocalyx may open a new issue to preserve the functions in cancer and infectious disease. The already used blockers of VEGF, a major permeability factor, may be enriched by new molecules that are easier to produce and cheaper. NO and PGI 2 can also be reconsidered as possible targets to therapy. Molecular imaging techniques, positron emission tomography, computed-tomography, and magnetic resonance imaging provided information that correlates with the markers of macrophages. Microvascularization and microvascular permeability correlated with 2-deoxy-2[8F] fluoroglucose uptake. These new techniques of imaging provide more precise detection of ongoing inflammation in human atheroma, a new strategy for vascular risk gradation, and new treatment [76].

One further step forward was achieved when we understood the regulation of permeability and relation between the EC molecular mechanism involved in the vascular complications of different diseases. The prevention of glycocalyx degradation can be one goal to achieve with new therapies.

Already known compounds, such as hydroxyethyl starch, have been shown to limit capillary leakage in acute respiratory distress syndrome [77]. 

Anti-PECAM antibodies have been proposed to regulate EC barrier function [78]. The inhibition of RhO A kinase may be an interesting approach to modulate EC barrier function. As we pointed out before, ROS formation inhibition or VEGF counteracting are two promising strategies to limit increased permeability and their consequences.
